# Hydroxyapatite Particles from Simulated Body Fluids with Different pH and Their Effects on Mesenchymal Stem Cells

**DOI:** 10.3390/nano11102517

**Published:** 2021-09-27

**Authors:** Hiroki Miyajima, Hiroki Touji, Kazutoshi Iijima

**Affiliations:** 1 Faculty of Engineering, Yokohama National University, 79-5 Tokiwadai, Hodogaya-ku, Yokohama 240-8501, Japan; miyajima-hiroki-dt@ynu.ac.jp; 2 Graduate School of Engineering Science, Yokohama National University, 79-5 Tokiwadai, Hodogaya-ku, Yokohama 240-8501, Japan; touji-hiroki-bh@ynu.jp

**Keywords:** hydroxyapatite, simulated body fluid, biomimetic synthesis, mesenchymal stem cells

## Abstract

Bone-like hydroxyapatite (HAp) has been prepared by biomimetic synthesis using simulated body fluid (SBF), mimicking inorganic ion concentrations in human plasma, or 1.5SBF that has 1.5-times higher ion concentrations than SBF. In this study, the controllable preparations of HAp particles from 1.5SBF with different pH values were examined. The particles obtained as precipitates from 1.5SBF showed different morphologies and crystallinities depending on the pH of 1.5SBF. Micro-sized particles at pH 7.4 of 1.5SBF had a higher Ca/P ratio and crystallinity as compared with nano-sized particles at pH 8.0 and pH 8.4 of 1.5SBF. However, a mixture of micro-sized and nano-sized particles was obtained from pH 7.7 of 1.5SBF. When Ca^2+^ concentrations in 1.5SBF during mineralization were monitored, the concentration at pH 7.4 drastically decreased from 12 to 24 h. At higher pH, such as 8.0 and 8.4, the Ca^2+^ concentrations decreased during pH adjustment and slightly decreased even after 48 h. In this investigation at pH 7.7, the Ca^2+^ concentrations were higher than pH 8.0 and 8.4.Additionally, cytotoxicity of the obtained precipitates to mesenchymal stem cells was lower than that of synthetic HAp. Controllable preparation HAp particles from SBF has potential applications in the construction of building components of cell scaffolds.

## 1. Introduction

Bone tissue is an organic–inorganic hybrid mainly composed of collagen and calcium phosphate, such as hydroxyapatite (HAp, Ca_10_(PO_4_)_6_(OH)_2_) [1]. Because HAp shows a high affinity to bone tissue [2], it has been clinically used as a bone substitute for large bone defects [3]. Recently, HAp cell scaffolds, embedded with mesenchymal stem cells (MSCs) for implantation, were developed because their osteogenic differentiation was promoted by HAp [4,5]. Furthermore, HAp can be widely used as a research tool, such as cell scaffolds mimicking the bone environment in biomedical research fields [6,7,8,9].

HAp can be prepared using various methods: hydrothermal synthesis [10], a wet chemical process [11], a sol-gel process [12], biogenic synthesis [13], and biomimetic synthesis [14,15,16]. Biological properties, such as cell adhesion and osteoinductivity, are affected by preparative methods because of differences in chemical and physical characteristics [17]. Therefore, preparative methods for various types of HAp particles have significant potential for the construction of cell scaffolds with different biological properties. In the biomimetic synthesis of the HAp inspired biomineralization process, simulated body fluid (SBF), a solution mimicking inorganic concentrations of human plasma, has been used [18,19]. In this method, HAp can be easily obtained under low-energy consumption and less environmental burden. At the initial stage of HAp formation in SBF, homogeneous nucleation of calcium phosphate triggers the growth of HAp crystals, and thus, the HAp crystal forms [20]. Furthermore, the HAp obtained from SBF is called bone-like HAp, showing similar inorganic composition to natural bone, which is different from the synthetic stoichiometric HAp [21]. Regarding the calcium/phosphorus (Ca/P) ratio, which was calculated by comparing calcium with phosphorus, bone-like HAp obtained from SBF showed a lower Ca/P ratio than commercially available stoichiometric HAp (Ca/P = 1.67) [22]. The low Ca/P ratio of bone-like HAp reflects the defective structure and low crystallinity of HAp. Previous reports have demonstrated high connectivity to bone tissues [23] and high resorption by osteoclasts [24] of bone-like HAp. These features suggest that bone-like HAp shows potential for attachment to bone tissue, followed by the substitution of a transplant with autologous bone.

To further promote HAp deposition, various types of modified SBFs have been developed. In particular, a supersaturation of SBF, 1.5SBF, which is a solution containing 1.5-fold higher ion concentration than that of the original SBF, has been prepared [25,26]. In 1.5SBF, nucleation and crystal growth of HAp are accelerated, facilitating the formation of HAp particles [25,26]. Another strategy is to control the solution pH of SBF [27]. The pH of SBF affects the yields and morphologies of HAp. Hashizume et al. demonstrated that smaller particles were mainly formed when the pH of 1.5SBF increased from 7.4 to 8.4 or 8.6 [27]. Although it is believed that the yields of HAp increase at a higher pH owing to a decrease in the solubility of HAp, few studies have been conducted on the effects of the pH of a solution on HAp formation. It appears that these morphological changes occurred in the SBF under weak alkaline conditions at an approximate pH value of 8.0 [27].

In this study, four types of 1.5SBF with pH values of 7.4, 7.7, 8.0, and 8.4 were prepared to elucidate the effect of the solution pH in alkali conditions on HAp formation from SBF. The morphologies and characterization of the precipitates obtained from the 1.5SBF were investigated using scanning electron microscopy (SEM), transmission electron microscopy (TEM), Fourier transform infrared (FT-IR) spectroscopy, and X-ray diffraction (XRD). To further understand the mechanism underlying HAp formation from SBFs with different pH values, calcium ion concentrations in 1.5SBF during the mineralization process were monitored by inductively coupled plasma atomic emission spectroscopy (ICP-AES) because calcium ions are important in facilitating the process of HAp nucleation and crystal growth. For future application of obtained precipitates from 1.5SBF for cell scaffolds, cytotoxicity of the precipitates to MSCs was also evaluated.

## 2. Materials and Methods

### 2.1. Reagents

All reagents were purchased from Nacalai Tesque, Inc. (Kyoto, Japan) and used without further purification. Ultrapure water (18.2 MΩ cm) was prepared using a Direct-Q UV5 (Merck Millipore, Burlington, MA, USA). The 1.5SBF was prepared using the same procedure for preparing conventional SBF in the following literature [18].

### 2.2. Mineralization from 1.5SBF with Different pH

The pH of 2 L 1.5SBF in a polypropylene bottle (As One, Osaka, Japan) was adjusted to the desired value by adding tris(hydroxymethyl)aminomethane (Tris). The pH of the solution was monitored with a pH meter (F-71; Horiba, Ltd., Kyoto, Japan) while stirring. The 1.5SBF was prepared at a different pH and then incubated at 37 °C for 24 h. After the removal of a large volume of supernatant, the dispersion of the precipitates was poured into a centrifuge tube and then centrifuged at 4640× *g* for 5 min (Model 5200; KUBOTA Corp., Tokyo, Japan). After the supernatant was removed, the precipitates were washed with ultrapure water and freeze-dried with a lyophilizer (FDU-1200, Tokyo Rikakikai Co., Ltd., Tokyo, Japan).

### 2.3. Characterization of Precipitates

The morphologies of precipitates from 1.5SBF at different pH were observed by SEM (SU8010; Hitachi High-Technologies Corporation, Tokyo, Japan) employing an acceleration voltage of 4.0–10.0 kV and TEM (JEM-2100F; JEOL Ltd., Tokyo, Japan) operated at 200 kV. The particle size distribution of precipitates was calculated from SEM and TEM images using ImageJ [28]. FT-IR spectra of the precipitates were obtained by a single reflection attenuated total reflection (ATR) using an FT-IR 6200 equipped with ATR PRO450-S (JASCO Corporation, Tokyo, Japan). XRD was performed using a SmartLab II instrument (Rigaku, Tokyo, Japan) with Cu-Kα radiation (tube voltage: 40 kV, tube current: 45 mA, and step width: 0.02°).

### 2.4. Measurement of Calcium Ion Concentration

The concentration of calcium ions at a solution pH of 1.5SBF (pH 7.4) was increased by 0.1, i.e., from pH value of 7.4 to 8.4, by adding Tris, which was measured by ICP-AES (ICPE-9000; Shimadzu, Tokyo, Japan). Moreover, the concentrations of calcium ions in the 1.5SBF at pH of 7.4, 7.7, 8.0, and 8.4 during incubation at 37 °C were also measured with the ICP-AES. Each solution with a different pH and incubation time was poured into a centrifuge tube and then centrifuged at 4640× *g* for 5 min. After the dispersed precipitates in the supernatant were removed by filtration through a microfilter (pore size, 0.22 μm, Millipore), the clear filtrate was collected in a tube and diluted in ultrapure water. Once 10-fold diluted solutions were preserved at 4 °C before measurement, 500-fold diluted solutions were measured by ICP-AES.

### 2.5. Cell Culture

Immortalized human bone marrow-derived MSCs, UE7T-13 cells [29] (Japanese Collection of Research Bioresources (JCRB), Cell Bank, Osaka, Japan), were cultured in Dulbecco’s modified Eagle’s medium (D-MEM, Nacalai Tesque, Inc., Kyoto, Japan) supplemented with 10% fetal bovine serum (FBS, Thermo Fisher Scientific, Waltham, MA, USA) and 1% penicillin (100 U/mL)/streptomycin (100 μg/mL) solution (Nacalai Tesque, Inc., Kyoto, Japan).

To prepare suspension of precipitates in culture medium, precipitates obtained from 1.5SBF or commercially available synthetic HAp particles (Sigma Aldrich, St. Louis, MO, USA) were dispersed in culture medium. The suspension of precipitates was sterilized by UV irradiation (254 nm, 4.9 W, 10 min). A total of 5000 cells/well were seeded into a 96-well plate (VIOLAMO, AS ONE Corporation, Osaka, Japan) and cultured for 24 h at 37 °C under a humidified 5% CO_2_ atmosphere. Then, the culture medium was replaced with prepared suspension of the precipitates, and cells were cultured for 24 h. After removal of the suspension of precipitates, cells were rinsed three times with D-PBS(-). Cell viabilities were determined by WST-8 assay with a CCK-8 cell counting kit (Dojindo, Kumamoto, Japan) according to manufacturer’s protocol. The culture medium (100 μL) was poured into the well, and then the CCK-8 reagent (10 μL) was added to the well. After 1 h, the solution was collected into a tube, and centrifugation (4640× *g*, 5 min) was carried out for removal of the remaining precipitates. The supernatant (100 μL) was poured into the 96-well plate, and absorbance at 450 nm was measured with a microplate reader (Thermo Fisher Scientific, Waltham, MA, USA) to calculate the cell viabilities.

## 3. Results

### 3.1. Mineralization from 1.5SBF with Different pH

**Figure 1:**
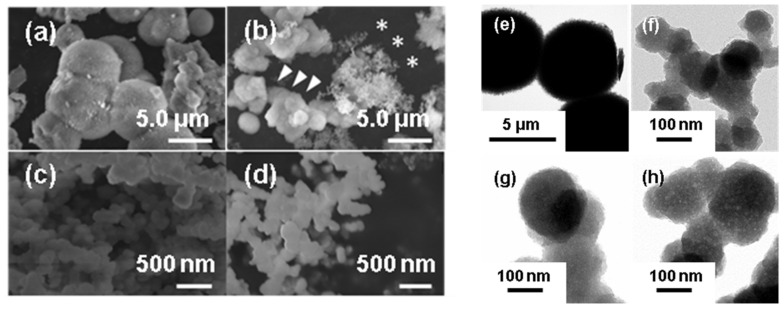
SEM images of the precipitates from 1.5SBF at pH 7.4 (**a**), pH 7.7 (**b**), pH 8.0 (**c**), and pH 8.4 (**d**). Arrowheads and asterisks indicate micro- and nano-sized particles, respectively. TEM images of the precipitates from 1.5SBF at pH 7.4 (**e**), pH 7.7 (**f**), pH 8.0 (**g**), and pH 8.4 (**h**).

**Figure 2:**
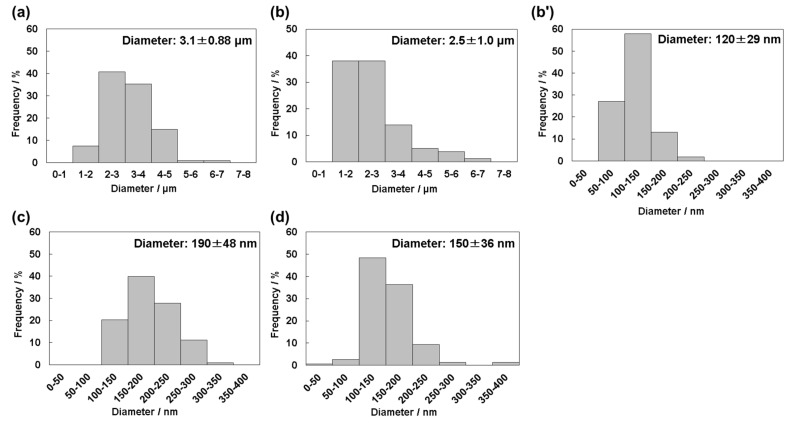
Particles size distribution for precipitates from 1.5SBF at pH 7.4 (**a**), pH 7.7 (micro-sized particles, (**b**), nano-sized particles, (**b’**)), pH 8.0 (**c**), and pH 8.4 (**d**). Diameters of particles were measured from SEM and TEM images.

When the pH of 1.5SBF was increased using Tris, the solution became cloudy at pH 8.0 and pH 8.4. After 24 h of incubation at 37 °C, precipitates were generated from all SBFs (pH 7.4, 7.7, 8.0, and 8.4). Yields of precipitates from 500 mL 1.5SBF, based on stoichiometric HAp (Ca_10_(PO_4_)_6_(OH)_2_), were 64.9 mg (51.2%; pH 7.4), 36.0 mg (28.4%; pH 7.7), 47.4 mg (37.4%; pH 8.0), and 95.4 mg (75.3%; pH 8.4), respectively. SEM images of the precipitates are shown in Figure 1. Precipitates from 1.5SBF at pH 7.4 were micro-sized particles with rough surfaces (Figure 1a), whereas those at pH 8.0 and 8.4 were nano-sized particles with smooth surfaces (Figure 1c,d). At a pH value of 7.7, both micro-sized and nano-sized particles were observed (Figure 1b). Detailed morphologies of the deposits were observed by TEM (Figure 1e–h). Micro-sized particles obtained from 1.5SBF at pH 7.4 had a plate-like structure (Figure S1). Nano-sized particles obtained from 1.5SBF at pH 7.7, 8.0, and 8.4 had heterogeneous inner structures (Figure 1f–h). The distribution of diameters measured from SEM and TEM images of the particles obtained in each condition are shown in Figure 2. Average and standard deviation were as follows: 3.1 ± 0.88 μm (pH 7.4), 2.5 ± 1.0 μm (pH 7.7, micro-sized particles), 120 ± 29 nm (pH 7.7, nano-sized particles), 190 ± 48 nm (pH 8.0), and 150 ± 36 nm (pH 8.4).

### 3.2. Characterization of Precipitates from 1.5SBF with Different pH

**Figure 3:**
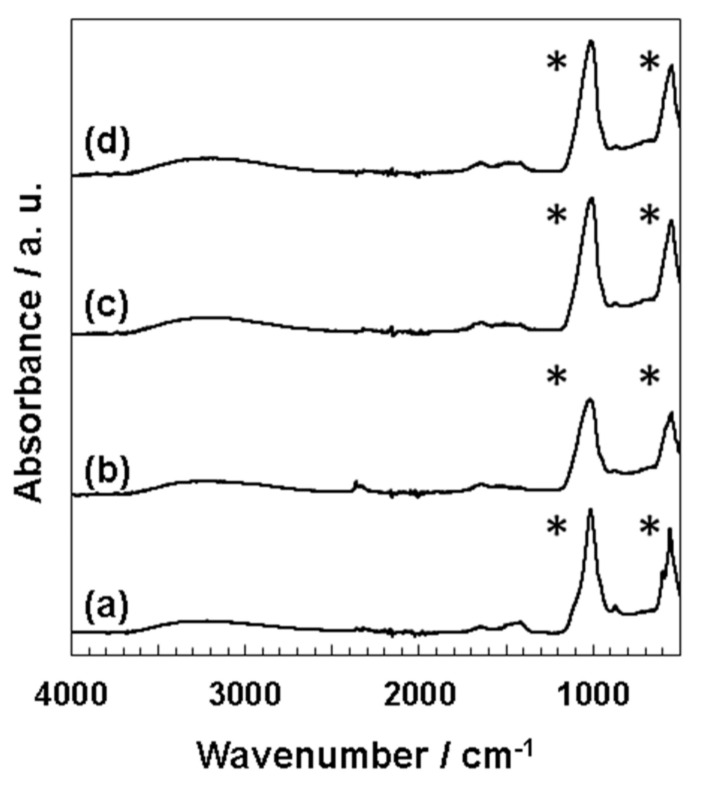
FT-IR spectra of the precipitates from 1.5SBF at pH 7.4 (**a**), pH 7.7 (**b**), pH 8.0 (**c**), and pH 8.4 (**d**). The asterisks indicate peaks assignable to stretching vibrations (*ν_3_*, 1200–1000 cm^−1^) and bending vibrations (*ν_4_*, 650–500 cm^−1^) of PO_4_^3−^.

EDX measurements equipped with SEM indicated that all deposits contained calcium and phosphorus (Figure S2a–d). The calculated Ca/P ratios were as follows: 1.66 (pH 7.4), 1.69 (pH 7.7, micro-sized particles), 1.50 (pH 7.7, nano-sized particles), 1.55 (pH 8.0), and 1.47 (pH 8.4). In addition to calcium and phosphorus, magnesium and sodium were detected in all samples. Cation (sum of Ca, Na, and Mg)/P ratios were also calculated at 1.83 (pH 7.4), 1.97 (pH 7.7, micro-sized particles), 1.69 (pH 7.7, nano-sized particles), 1.82 (pH 8.0), and 1.71 (pH 8.4). FT-IR spectra of the precipitates obtained from four types of 1.5SBF (pH = 7.4, 7.7, 8.0, 8.4) showed that two peaks were mainly detected in the range of 1150–900 and 650–500 cm^−1^, being assignable to the stretching and bending vibrations of phosphate groups (PO_4_^3−^), respectively (Figure 3) [7]. Peaks detected in precipitates at pH 7.4 were sharper than those at pH 7.7, 8.0, and 8.4. Broad peaks at 3600–3000 cm^−1^ and weak peaks at 1500–1400 cm^−1^ were also seen from all four kinds of FT-IR spectra, indicating the presence of hydroxyl groups (OH) and carbonate species (CO_3_^2−^), respectively.

**Figure 4:**
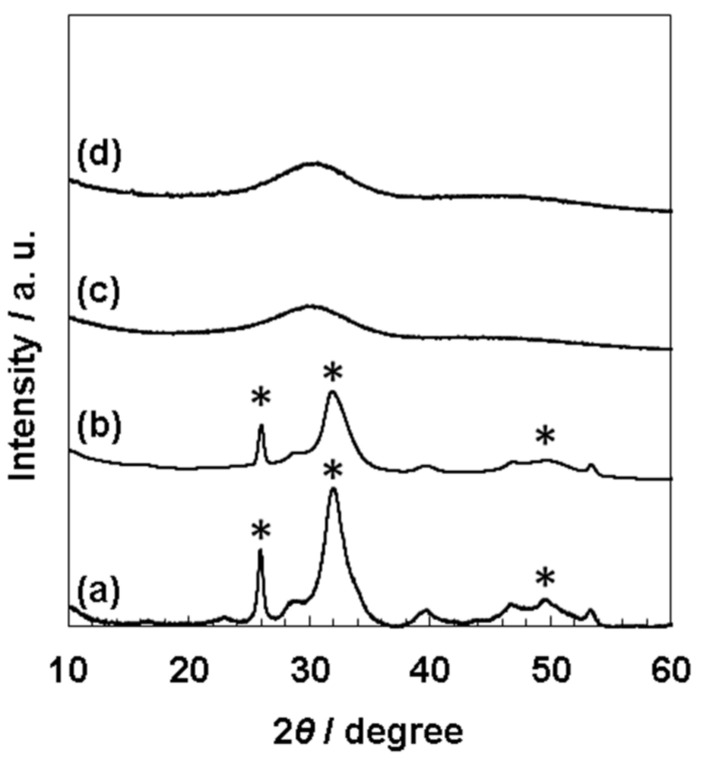
X-ray diffraction (XRD) pattern of the precipitates from 1.5SBF at pH 7.4 (**a**), pH 7.7 (**b**), pH 8.0 (**c**), and pH 8.4 (**d**). The asterisks show peaks corresponding to typical peaks of HAp.

In the XRD measurement (Figure 4), the results at pH 7.4 and pH 7.7 (Figure 4a,b) showed the strong peaks at 2θ = 26°, 32°, and 49° in accordance with the reported peaks with high intensity [30]. These peaks correspond to the (002) planes at 25.9°, (211) at 31.8°, (112) at 32.2°, (300) at 32.9°, and (213) at 49.5° [30]. The weak peaks with low intensity were also observed at 39°, 47°, 50°, assigned to (212) at 39.2°, (310) at 39.8°, (222) at 46.7°, and (321), (410), (402), and (004) at 50–53° [30]. However, broad peaks were only detected at approximately 30° from the samples at pH 8.0 and 8.4 (Figure 4c,d), indicating an amorphous state [27].

### 3.3. Effect of pH of 1.5SBF on Calcium Ion Concentrations

**Figure 5:**
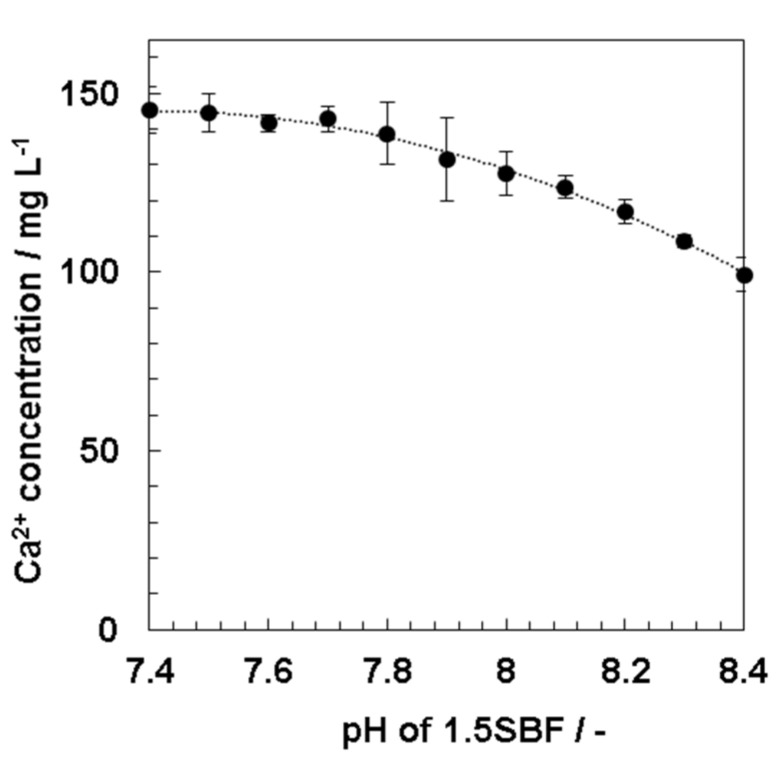
Calcium ion concentration in 1.5SBF by increasing pH of the 1.5SBF.

Calcium ion concentrations in 1.5SBF were measured with ICP-AES when the prepared solution pH of 1.5SBF (pH 7.4) at 37 °C was increased by 0.1 from pH 7.4 to pH 8.4 with Tris. Results showed that the calcium ion concentrations gradually decreased from 146 mg/L at pH 7.4 to 99.3 mg/L at pH 8.4 (Figure 5).

### 3.4. Calcium Ion Concentration Change in 1.5SBF during Mineralization

**Figure 6:**
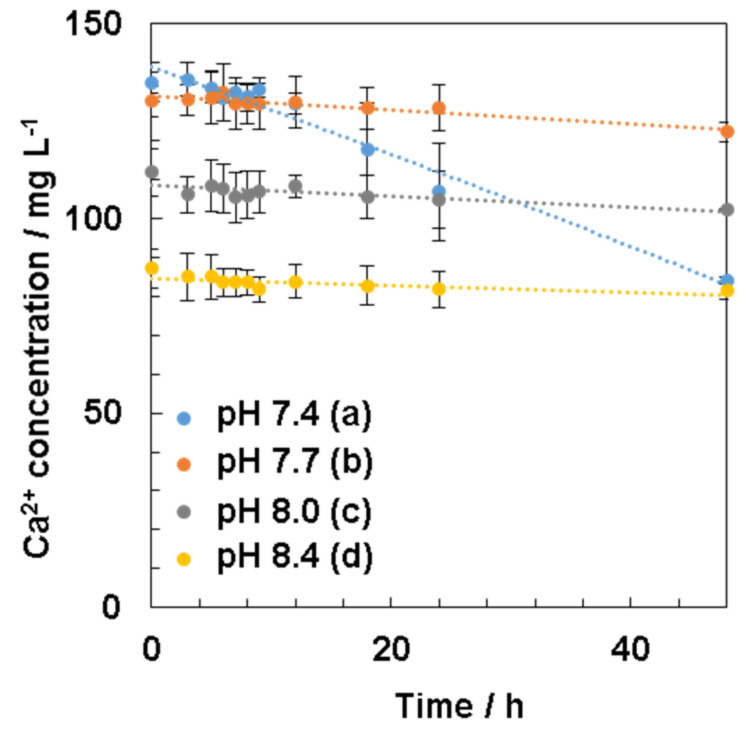
Calcium ion concentration in 1.5SBF during formation of the precipitates in 1.5SBF at pH 7.4 (**a**), pH 7.7 (**b**), pH 8.0 (**c**), and pH 8.4 (**d**).

For further understanding the HAp nucleation process affected by the solution pH, the calcium ion concentrations in four types of 1.5SBF (pH = 7.4, 7.7, 8.0, and 8.4) during incubation were measured for 48 h (Figure 6).

Consequently, the initial calcium ion concentrations were different for all four types of 1.5SBF (pH = 7.4, 7.7, 8.0, and 8.4) (Figure 6); for example, 135 mg/L (pH 7.4) and 87.2 mg/L (pH 8.4). When 1.5SBF was incubated, the calcium ion concentrations in all types of 1.5SBF gradually decreased over time as compared with initial concentrations, especially the concentrations at pH 7.4, which significantly decreased from 12 to 24 h. However, at higher pH, such as pH 7.7, 8.0, and 8.4, calcium ion concentrations decreased slightly as compared with pH 7.4.

### 3.5. Cytotoxicity of Precipitates

**Figure 7:**
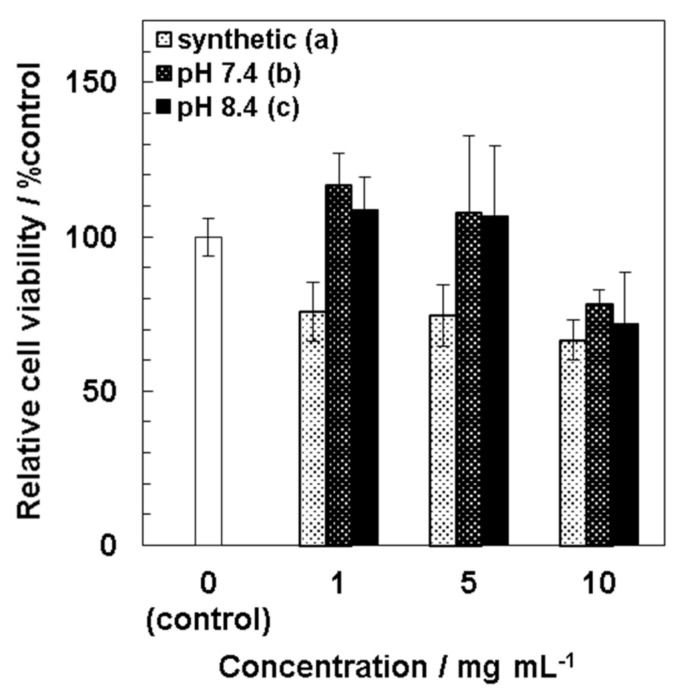
Viability of UE7T-13 cells cultured in suspension of synthetic hydroxyl apatite and precipitates obtained from 1.5SBF for 24 h (*n* = 3). Synthetic HAp particles (**a**), microparticles obtained from 1.5SBF at pH 7.4 (**b**), and nanoparticles obtained from 1.5SBF at pH 8.4 (**c**).

Cytotoxicity of precipitates to MSCs was evaluated by the WST-8 assay (Figure 7). At lower concentrations at 1–5 mg/mL, both kinds of precipitates obtained from 1.5SBF at pH 7.4 and pH 8.4 showed lower cytotoxicity to MSCs than synthetic HAp particles. Viabilities of MSCs exposed to synthetic HAp particles (1–10 mg/mL) were 65–75%. At a higher concentration such as 10 mg/mL of the precipitates, viabilities of MSCs were deceased as much as synthetic HAp particles.

## 4. Discussion

In this study, we demonstrated that precipitates from four types of 1.5SBF (pH = 7.4, 7.7, 8.0, 8.4) showed different morphologies, such as particle size and surface structure. From pH 7.4 of 1.5SBF, micro-sized particles (diameter: 3.1 ± 0.88 μm, Figure 2a) with a plate-like structure were obtained (Figure 1a,e). The Ca/P ratios of the deposits from pH 7.4 were 1.66 (SEM-EDX, Figure S2a) and 1.60 (TEM-EDX, Supplemental Figure S3a). The Ca/P ratio, which is slightly lower than the theoretical value of 1.67, and the presence of magnesium and sodium in SEM-EDX (Figure S2a), suggested that the precipitates were ion-substituted HAp, whose calcium sites were slightly substituted by magnesium and sodium [27]. Calculated cation (Ca, Mg, and Na)/P ratios of precipitates were higher than the theoretical value (1.67). It may be inferred that phosphate anion sites of precipitates were also substituted with other anion species. In the FT-IR spectra (Figure 3), the peaks corresponding to carbonate or hydroxyl group were detected. Although the ratio of anion species containing precipitates could not be determined, all cation/anion ratios in precipitates may be closer to the theoretical value.

The splitting sharp peaks of the phosphate groups in the FT-IR spectra (Figure 3a) indicate the high crystallinity of the particles [27]. The XRD results also supported the high crystallinity of the precipitates from pH 7.4 of 1.5SBF. However, from 1.5 SBF, whose pH was 8.0 and 8.4, only nano-sized particles (190 ± 48 nm from pH 8.0 and 150 ± 36 nm from pH 8.4, Figure 2c,d) were formed (Figure 1c,d,g,h). For nano-sized particles, XRD showed only a broad peak at approximately 30° originating from the amorphous state [27] (Figure 4c,d). In the FT-IR spectra of the nano-sized particles, the peaks derived from the phosphate groups were broad (Figure 3). These results suggested that the nanoparticles showed lower crystallinity than the microparticles obtained at pH 7.4. Moreover, when pH of the 1.5SBF solution was 7.7, both micro- and nanoparticles with diameters of 2.5 ± 1.0 μm and 120 ± 29 nm, respectively, were obtained (Figure 2b,b’). SEM-EDX and TEM-EDX spectra indicated that the Ca/P ratio of the microparticles was higher than that of the nanoparticles (Figures S2 and S3). The Ca/P ratio of the microparticles and nanoparticles at pH 7.7 was similar to the ratio of microparticles obtained from 1.5SBF (pH 7.4) and nanoparticles obtained from 1.5SBF (pH 8.0 or 8.4) (Figures S2 and S3a–d). In the FT-IR spectra of precipitates at pH 7.7 (Figure 3b), the shapes of the peaks from the phosphate group were similar to those at pH 8.0 and 8.4. XRD diffraction showed peaks assignable to HAp, but not broad peaks originating from the amorphous state (Figure 4b). The XRD pattern is in rough agreement with the peaks of precipitates at pH 7.4 (Figure 4a). However, the peaks at pH 7.7 were not significantly sharp, especially at approximately 50° assignable to the (213) plane [30]. Spectroscopic analyses also indicated that the precipitates in pH 7.7 were a mixture of micro- and nano-particles.

For the investigation of the effect of the solution pH of 1.5SBF on HAp formation, the calcium ion concentrations in 1.5SBF, at the time when the pH was increased by 0.1 from 7.4 to 8.4, were measured. Calcium ion concentrations of 1.5 SBF at different pH reveal initial calcium ion concentrations before incubation for mineralization. The calcium ion concentrations gradually decreased with increasing solution pH (Figure 5). At approximately pH 8.0, the solution immediately became turbid, which may be owing to a decrease in the solubility of calcium phosphate by increasing the solution pH [31]. These results indicated that the formation of precipitates, such as calcium phosphate, occurred immediately after the solution pH was adjusted to 8.4. Because the solubility of calcium phosphate was lowered in supersaturated 1.5SBF with respect to HAp, calcium phosphate would be precipitated by increasing the solution pH.

The effect of the solution pH on HAp formation was investigated by changing the calcium ion concentrations in 1.5SBF with different pH values during incubation. The measured calcium ion concentrations approximately accorded with the calcium ion concentrations calculated from yields of precipitates from 1.5SBF (Table S1). From the results, the mechanism to form precipitates in 1.5SBF will be speculated. At pH 7.4 of 1.5SBF, the calcium ion concentration gradually decreased for 48 h (Figure 6a), in accordance with the literature [32]. The calcium ion concentrations measured by ICP-AES after 24 h incubation (107 mg/L) approximately corresponded to the values estimated from yields (98.8 mg/L). The obtained result at pH 7.4 indicates that homogeneous nucleation occurs, and the formed calcium phosphates, such as amorphous calcium phosphate (ACP), grow to HAp [20] by consuming the calcium ion concentration. However, at pH 8.0 and 8.4, calcium ion concentrations decreased immediately at the initial state, i.e., 0 h, and the concentrations were not significantly consumed even after 48 h (Figure 6c,d). On appearance, the solution became slightly cloudy after the solution pH was adjusted to pH 8.0 and 8.4. Comparing the difference in yields between pH 8.0 and 8.4, twice the amount of precipitates was obtained at pH 8.4. Because significant declines in calcium ion concentrations were not observed at either pH after incubation, these differences would depend on the solubility of calcium phosphate in the initial solution pH. The results indicated that precipitates at 0 h did not significantly grow and form complete HAp crystals. At pH values higher than 7.4, it was inferred that the crystal growth was inhibited. These results suggested that the initial decline in calcium ion concentration in 1.5SBF was critical at a higher pH, such as pH 8.0 and 8.4. At pH 7.7, the calcium ion concentration decreased slightly for 48 h (Figure 6b), as well as at pH 8.0 and 8.4; however, the concentration at 0 h was higher than at pH 8.0 and 8.4. This result suggested that ACP was generated by increasing the solution pH. Significantly, precipitation at pH 7.7 occurred slightly because pH 7.7 was not as high as at pH 8.0 and 8.4. Because microparticles were observed at pH 7.7, HAp nucleation would have occurred owing to the higher calcium ion concentration in the solution. The obtained results suggested that nanoparticles, such as ACP, were formed in the initial state, and that ACP would grow to microparticles during incubation.

From assessment of cytotoxicity of the particles to MSCs (Figure 7), it was found that chemically synthetic HAp particles showed cytotoxicity at 1 mg/mL, whereas precipitates obtained from 1.5SBF at pH 7.4 and pH 8.4 did not at 5 mg/mL. These results indicated that the precipitates from 1.5SBF showed lower cytotoxicity as compared with synthetic HAp particles. One possibility is ion-substitution of the particles contributing to lower toxic effects on MSCs. Another possibility is difference in sizes of particles. Cytotoxicity of the particles to MSCs would attribute to cellular uptake of the particles and subsequently increased intracellular calcium ion. Cellular uptake through endocytosis is generally increased by increasing the concentrations of nanoparticles and decreasing their particle sizes. From Figure 2 and Figure S4, diameters of precipitates obtained from 1.5SBF were 3.1 ± 0.88 μm (pH 7.4) and 150 ± 36 nm (pH 8.4), whereas that of synthetic HAp particles was 63 ± 25 nm. From obtained results, chemically synthetic HAp particles with smaller sizes showed significant cytotoxicity to MSCs. It has been known that concentrations, sizes, and morphologies of HAp particles are involved in cellular uptake [33,34]. HAp particles uptaken into cells would induce reactive oxygen species (ROS) generation, and oxidative stress would cause lysosomal rupture, mitochondria dysfunction, and DNA damage inducing apoptosis [35].

In this study, it was demonstrated that HAp particles with different morphologies and crystallinities can be easily prepared by controlling the pH of 1.5SBF. When HAp is used as a component of cell scaffolds, its chemical and physical properties, such as Ca/P ratio, crystallinity, or surface roughness, drastically affect the cells cultured in the scaffolds [17]. Fabrication using HAp powder was carried out by press [36] or using HAp cement [37]. Recently, studies have been conducted on the microfabrication of calcium phosphate. In these studies, cell scaffolds with designed complicated structures were fabricated using ceramic slurry containing resin dispersed calcium phosphate, such as HAp or β-tricalciumphosphate (β-TCP) [38,39,40], thus accomplishing more complex three-dimensional (3D) cell culture systems, different from ordinary two-dimensional cell culture. These techniques can artificially create a 3D biological environment that supports cellular functions, such as cell survival, cellular crosstalk, or differentiation. Combined with these fabrication techniques, controllable HAp preparation will facilitate the development of various types of created cell scaffolds.

## 5. Conclusions

Biomimetic synthesis of HAp using SBF was conducted. Precipitates were spontaneously formed in 1.5SBF, a solution having 1.5-times higher ion concentrations than SBF, and the morphologies and crystallinities of the precipitates were affected by the solution pH of 1.5SBF. The obtained precipitates at 1–5 mg/mL showed lower cytotoxicity to MSCs. These results would be helpful to understand the effect of solution pH of 1.5SBF on formation of particles. Since HAp can be applied for bone remodeling or bone substitutes in vivo, this method to easily obtain different kinds of HAp particles can contribute in preparing various types of cell scaffolds by surface modification of polymer scaffolds or construction of Hap-based three-dimensional structures for bone modeling.

## Supplementary Materials

The following are available online at https://www.mdpi.com/article/10.3390/nano11102517/s1, Figure S1: TEM images of the precipitates from 1.5SBF at pH 7.4. Scale bar: 200 nm (a), and 100 nm (b), Figure S2: SEM-EDX profiles of precipitates obtained from 1.5SBF at pH 7.4 (a), pH 7.7 (micro-sized particles, b, nano-sized particles, b’), pH 8.0 (c), and pH 8.4 (d), Figure S3: TEM-EDX spectra of precipitates obtained from 1.5SBF at pH 7.4 (a), pH 7.7 (nano-sized particles, b), pH 8.0 (c), and pH 8.4 (d), Figure S4: (a) TEM images of synthetic HAp particles. (b) Particles size distribution of chemically synthesized HAp particles. Diameters of particles were measured from TEM images, Table S1: Calcium ion concentration measured by ICP-AES and estimates from yield in each pH of 1.5SBF after 24-h incubation.
