# Supplementary material for: Hydroxyapatite Particles from Simulated Body Fluids with Different pH and Their Effects on Mesenchymal Stem Cells
Source: Nanomaterials (Basel). 2021 Sep 27;11(10):2517. doi: 10.3390/nano11102517 (PMC8538532; doi:10.3390/nano11102517)
Supplement: Supplementary file 1 [file nanomaterials-11-02517-s001.zip › nanomaterials-1375719-supplementary.pdf]

nanomaterials

Supplementary Materials

Hydroxyapatite Particles from Simulated Body Fluids with Different pH and Their Effects on Mesenchymal Stem Cells

Hiroki Miyajima [1], Hiroki Touji [2] and Kazutoshi Iijima [1,*]

[1] Faculty of Engineering, Yokohama National University, 79-5 Tokiwadai, Hodogaya-ku, Yokohama 240-8501, Japan; miyajima-hiroki-dt@ynu.ac.jp
[2] Graduate School of Engineering Science, Yokohama National University, 79-5 Tokiwadai, Hodogaya-ku, Yokohama 240-8501, Japan; touji-hiroki-bh@ynu.jp
* Correspondence: iijima-kazutoshi-mh@ynu.ac.jp; Tel.: +81-45-339-3997

1. Characterization of precipitates from 1.5SBFs

Surface of micro-sized particles obtained from 1.5SBF at pH 7.4 were observed by transmission electron microscopy (TEM) (Figure S1). Energy dispersive X-ray (EDX) spectra of precipitates were obtained using Esprit 1.9 (Bruker Nano GmbH, Berlin, Germany) with scanning electron microscopy (SEM) (SU8010) (Figure S2) and JED-2300T (JEOL Ltd., Tokyo, Japan) equipped with TEM (JEM-2100F) (Figure S3). Ca/P ratios were calculated from the detected results of peaks.

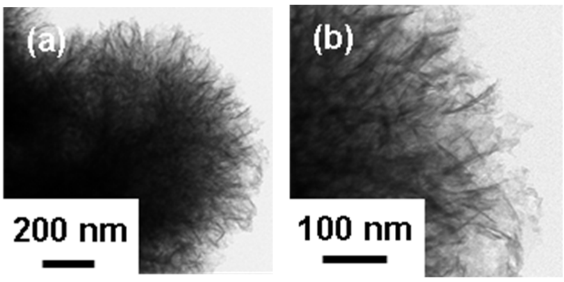

Figure S1. TEM images of the precipitates from 1.5SBF at pH 7.4. Scale bar: 200 nm (**a**), and 100 nm (**b**).

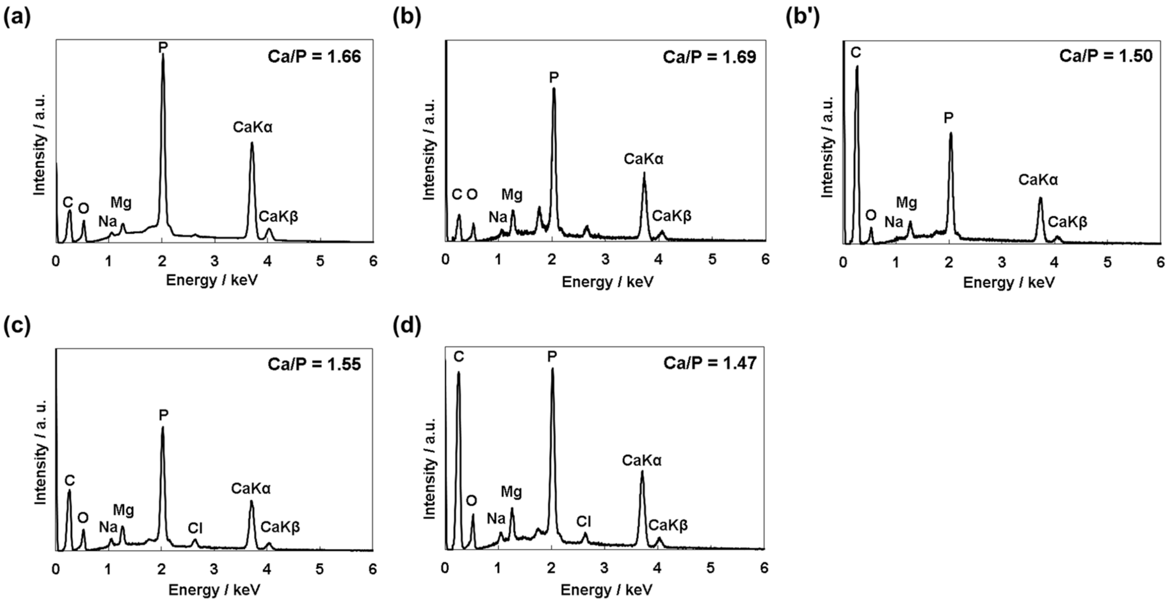

Figure S2. SEM-EDX profiles of precipitates obtained from 1.5SBF at pH 7.4 (**a**), pH 7.7 (micro-sized particles, (**b**), nano-sized particles, (**b'**), pH 8.0 (**c**), and pH 8.4 (**d**).

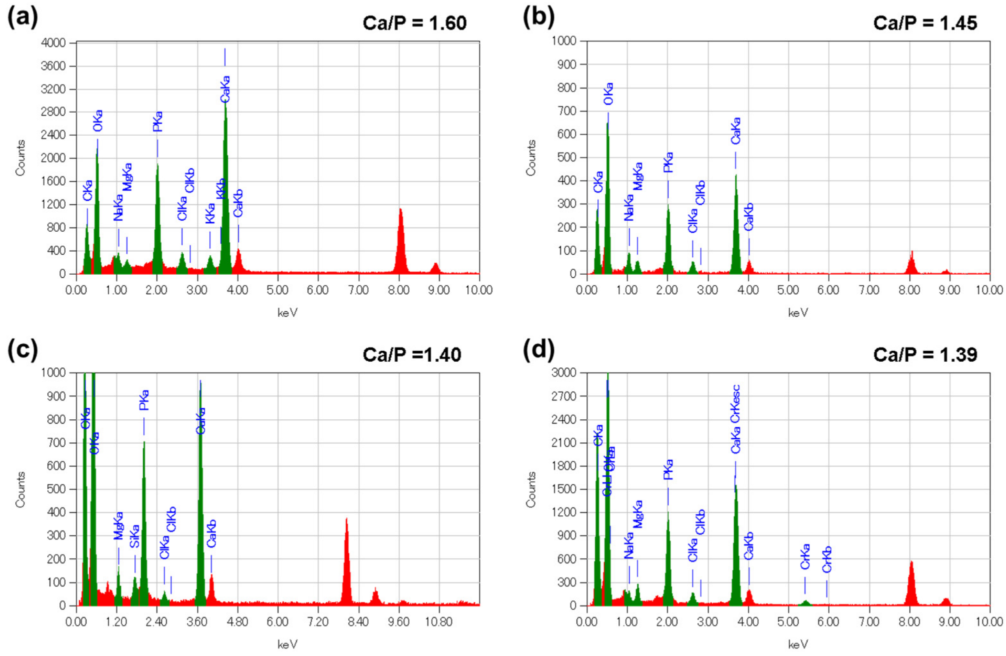

Figure S3. TEM-EDX spectra of precipitates obtained from 1.5SBF at pH 7.4 (**a**), pH 7.7 (nano-sized particles, **b**), pH 8.0 (**c**), and pH 8.4 (**d**).

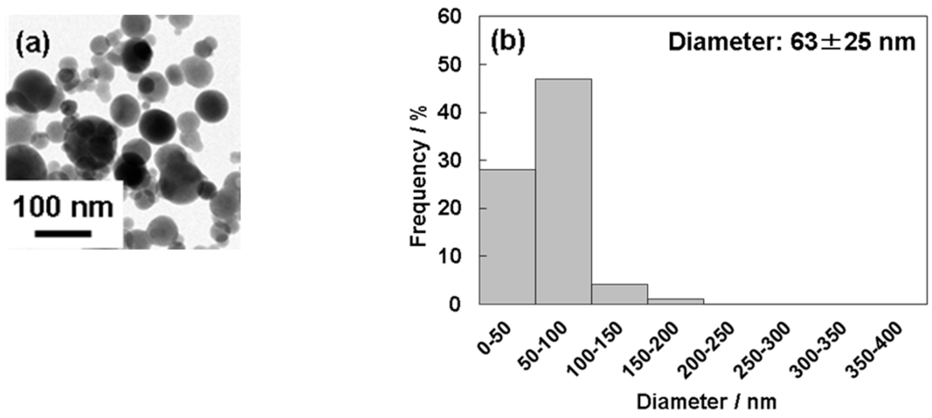

Figure S4. (**a**) TEM images of synthetic HAp particles. (**b**) Particles size distribution of chemically synthesized HAp particles. Diameters of particles were measured from TEM images.

Table S1. Calcium ion concentration measured by ICP-AES and estimates from yield in each pH of 1.5SBF after 24-h incubation.

pH	Measured $[Ca^{2+}]$ from ICP / mg L^{-1}	Estimated $[Ca^{2+}]$ from yield / mg L^{-1}
7.4	43.2	51.2
7.7	21.5	28.4
8.0	45.0	34.7
8.4	68.2	75.4
